# Prevalence of oncogenic human papillomavirus in pregnant adolescents, association with colpocytological changes, risk factors and obstetric outcomes

**DOI:** 10.1016/j.clinsp.2022.100127

**Published:** 2022-10-31

**Authors:** Henrique Diório de Souza, Adriana Lippi Waissman, Giselle Rodrigues Mota Diório, Stela Verzinhasse Peres, Rossana Pulcineli Vieira Francisco, Marco Aurélio Knippel Galletta

**Affiliations:** a Disciplina de Obstetrícia, Departamento de Obstetrícia e Ginecologia, Faculdade de Medicina, Universidade de São Paulo (FMUSP), São Paulo, SP, Brazil; b Departamento Materno Infantil, Faculdade de Medicina, Universidade Federal de Juiz de Fora, Juiz de Fora, Minas Gerais, MG, Brazil; c Divisão de Clínica Obstétrica, Hospital das Clínicas da Faculdade de Medicina da Universidade de São Paulo (HCFMUSP), São Paulo, SP, Brazil; d Departamento de Internato, Faculdade de Medicina, Universidade Federal de Juiz de Fora, Juiz de Fora, Minas Gerais, MG, Brazil

**Keywords:** Papillomavirus infections, Papanicolaou test, Pregnancy in adolescence, Prenatal care, HPV, human papillomavirus, HC, hybrid capture, DNA, deoxyribonucleic acid, OR, odds ratio, IBM, international business machines, SPSS, statistical package for the social sciences, NY, New York, CI, confidence interval, USD, United States Dollar, HSIL, squamous intraepithelial lesion, LSIL, low-grade squamous epithelial lesion, ASC-US, atypical squamous cells of undetermined significance, BMI, body mass index, pH, cologarithm of hydronium ion activity

## Abstract

**Purpose:**

The authors aim to carry out an investigation on the impact of cervical oncogenic Human Papillomavirus (HPV) detection in pregnant adolescents, to clarify the prevalence and risk factors, considering the importance and lack of data on this topic in Brazil.

**Methods:**

A cross-sectional study was conducted with adolescents receiving prenatal care in a tertiary hospital in São Paulo, Brazil, with routine Pap smear and oncogenic HPV detection test in the uterine cervix. The authors sought to associate the results of these tests with demographic and obstetric variables.

**Results:**

A total of 303 pregnant adolescents whose mean age was 15.30 ± 1.22 years comprised the study subjects. The frequency of high-risk HPV cervical detection was 50.50%. Multivariate analysis revealed that a large number of partners in their lifetime (OR = 1.27) and having a religion (OR = 2.05) were risk factors for cervical detection of oncogenic HPV, while schooling appeared as a protective factor (OR = 0.85). There was an association between this detection and colpocytological alterations, reaching almost 30% of patients, but without association with obstetric and neonatal outcomes.

**Conclusion:**

The prevalence found is one of the highest in Brazil and worldwide. A greater number of partners during their lifetime and having religion were detected as possible factors associated with cervical HPV detection. Detection of cervical HPV-DNA did not influence obstetric and neonatal outcomes. The findings of this study reinforce the need to implement educational measures capable of modifying the incidence of sexually transmitted infections in this population and capable of promoting adherence to HPV vaccination programs.

## Highlights

• One out of every two pregnant adolescents (50.5%) followed in prenatal care were positive for high-risk HPV and about 1 in 6 pregnant women (17.5%) had an abnormal Pap smear.
• Positivity for oncogenic HPV was an important risk factor for colpocytological alterations, especially for Low-grade Squamous epithelial Lesion (LSIL), increasing the risk by nine times.
• Higher education, fewer lifetime partners, and profession of religion were associated with the diagnosis of oncogenic HPV in pregnancy.

## Introduction

The Human Papillomavirus (HPV) is a nonenveloped virus with an icosahedral capsid and a double-stranded DNA that induces intense cell proliferation. More than 120 types of HPV have been identified and approximately half of them infect the genital tract. They can be divided into low-risk and high-risk viruses according to their ability to cause oncogenic cytological abnormalities in the uterine cervix. Among the high-risk oncogenic viruses, the authors would like to mention the following subtypes: 16, 18, 31, 33, 35, 39, 45, 51, 52, 56, 58, 59, 67, 68, and 70.^1^^,^^2^

The cytological changes engendered by the Human Papillomavirus (HPV) were first reported in 1956.^3^ Today HPV is acknowledged as a necessary cause for the development of uterine cervix neoplasm. Cervical cancer ranks fourth among the cancers and it is the neoplasm with the largest incidence in women worldwide, except for the nonmelanoma skin neoplasms.^4^ In 2017 it accounted for 6385 deaths in Brazil, corresponding to 1.11% of the deaths of the female sex. In 2020 it was estimated that 16,590 new cases would occur, corresponding to 15.43 cases per 100,000 Brazilian women.^5^

The HPV-caused infections tend to clear within 9 to 15 months. Nevertheless, the infection may lie dormant in some women, and persist in a minority.^6^ The independent risk factors associated with an HPV infection include the following: low educational level, first sexual intercourse at an early age, multiple sexual partners, multiple deliveries, smoking, patients younger than 30 years, alcohol consumption, partner belonging to an older age bracket, singlehood and the infrequent use or the absence of the use of barrier methods.^7^, ^8^, ^9^, ^10^, ^11^, ^12^, ^13^, ^14^ Prevalence of HPV in the uterine cervix of women, in general, varies from 7.30% to 74.60%,^15^^,^^16^ whereas prevalence among adolescents is reported to be between 15% and 90%.^17^^,^^18^ On the other hand, the prevalence of oncogenic HPV among adult women in general ranges from 5.30% to 54.30% in the literature,^15^^,^^16^ whereas among adolescents the figures fall between 10.74% and 51.70%.^19^^,^^20^ Some recent evidence appears to indicate that pregnancy has no bearing on prevalence, incidence, or HPV clearance.^21^ However, other references state that hormonal and immunological changes related to pregnancy are associated with the activation of cervical HPV infection, increasing the risk of cervical dysplasia.^21^^,^^22^

The existence of evidence on a possible association between the gestational period and cervical HPV infection, as well as the existence of references informing about a higher rate of HPV isolation in young populations, motivated this study.^10^^,^^22^^,^^23^ There are few studies conducted with pregnant women to evaluate the high-risk HPV-caused infection of the uterine cervix. Research tends to be even scarcer when it comes to pregnant adolescents. Therefore, this study aimed to determine the frequency of occurrence and the risk factors associated with a diagnosis of cervical HPV in pregnant adolescents treated at an outpatient clinic for specialized prenatal care in a tertiary hospital in São Paulo, Brazil.

## Materials and methods

This observational cross-sectional study was carried out in a tertiary teaching hospital, the Hospital das Clínicas da Faculdade de Medicina da Universidade de São Paulo. The hospital is located in the city of São Paulo, the most populous metropolis in Brazil.

An analysis was performed of the medical and laboratory data of all of the pregnant adolescents aged 10 through 18 years admitted to the outpatient clinic for the prenatal care of pregnancy in the adolescence of the Obstetric Clinic of the aforementioned hospital from January 01, 2010, to January 01, 2016. Excluded from the study were the pregnant adolescents who did not show up for prenatal care before the collection of vaginal smears, the adolescents with insufficient information in their medical records, or those with no access to it. Sociodemographic characteristics, clinical features, laboratory data, pathological history, obstetric history, and obstetric and neonatal outcomes were retrieved from the database of the sector. The adolescents’ gestational ages were estimated using the first day of the last menstruation or the first ultrasound exam during prenatal care.

The collection of cervical cytology and the performance of hybrid capture for HPV-DNA in pregnant adolescents are part of the protocols of the hospital where the study was carried out, although official Brazilian guidelines do not recommend performing cytology before the age of 25 years.

The data from 404 pregnant adolescents were analyzed. Four of the patients were excluded given the lack of access to their medical histories, and 97 of them were because they missed the Pap smear test, resulting in 303 cases for analysis.

### Collection of material from the uterine cervix

Investigation of HPV in the uterine cervix was usually performed during the first prenatal visit. The participants were subjected to a speculum examination for collecting cells from the ectocervix and the initial quarter of the endocervical canal with the aid of an Ayre spatula or a brush. The material that was gathered was transferred to a glass slide for an oncotic colpocytology (Pap smear) test and to a specific liquid medium from the *digene* HC2 DNA Collection Device for detection of high-risk HPV. The search for HPV types was conducted with the *digene* HC2 High-Risk HPV DNA test using the Hybrid Capture® 2 (HC2) technology. The test is based on hybrid-capture strategies and the use of probes for detecting 13 viral subtypes of HPV (16, 18, 31, 33, 35, 39, 45, 51, 52, 56, 58, 59, and 68).

### Data management and statistical analysis

A database was built with Microsoft Excel for Office 365 software. Statistical analyses were performed with the IBM SPSS Statistics for Windows 20.0 (IBM Corp., Armonk, NY) software.

Qualitative data were reported as measures of absolute and relative frequency (percentage) and compared by means of the chi-square test or the Fisher exact test as appropriate. Quantitative data were displayed as measures of central tendency (mean, median) and measures of dispersion (standard deviation). They were initially tested for normality of distribution with the Kolmogorov-Smirnov tests. Those with normal distribution had their means compared through the Student *t*-test. The medians of the nonparametric data were compared with the Mann-Whitney tests. The binary logistic regression was used to evaluate the associations between the variables predictive of an outcome with HPV present. The magnitude of the association was estimated by calculating the Odds Ratio (OR) and its respective 95% Confidence Interval (95% CI). Significant variables, those with a p-value lower than 0.15 (p < 0.15) in the univariate analysis and those of possible clinical importance were included in the multivariate regression model using the stepwise method. The goodness of fit of the logistic regression model was assessed through the Hosmer-Lemeshow test by seeking p values higher than 0.80 (p > 0.80). Associations with p values lower than 0.05 (p < 0.05) were deemed statistically significant.

### Ethics

The Research Ethics Committee of the Hospital das Clínicas da Faculdade de Medicina da Universidade de São Paulo approved the present study (presentation certificate for ethical appraisal number 65511617.0.0000.0068). An informed consent statement was waived because the project was a retrospective study with data retrieved from the patient's medical histories and there was no direct contact with the patients. All precautions were taken to protect the privacy of the research subjects and the confidentiality of personal information. The hospital's database identifies patients by numbers. Soon, the names of the women became anonymous to the researchers.

## Results

Of the 303 pregnant adolescents included in the study, 153 (50.50%) tested positive for high-risk HPV, and 150 tested negative. An overall analysis of the group showed that the mean age of the participants was 15.30 ± 1.22 (range: 11‒18 years) and the average number of years of schooling was 9.08±1.79 (range: 4‒14 years). Most of the adolescents (83.50%) were married or cohabited or were single but had a partner. The partners’ mean age was 20.40 (range: 13‒47 years). A large proportion of the adolescents was Catholic (44.37%) or Evangelical (33.44%) whose monthly family income was less than 5 Brazilian minimum salaries, that is, less than USD 1500 per month (55%). Of those stating they were religious, only 42.21% (103/244) declared they actually practiced their religion.

At the beginning of prenatal care, 10.56% reported the habit of alcohol consumption and another 10.56% declared they had the habit of smoking. Also, 7.92% stated they used or had used marijuana and 1.32% cocaine. Most adolescents had never been to a gynecologist before pregnancy (53.14%), and more than two-thirds were using some kind of contraceptive method (73.93%), mainly the male condom (45.36%) when they became pregnant. Adolescents aged over 14 years had a higher frequency of visits to the gynecologist before pregnancy than pregnant women aged less than or equal to 14 years (p < 0.001, OR = 2.91, 95% CI 1.65‒5.13). The mean sex frequency (per week) was 1.74 ± 1.80 and the mean time span of sexual activity (in months) prior to the pregnancy was 17.98 ± 13.14 (range: 1‒84). The average number of partners up to the time of the interview was 1.82 ± 1.64 (range: 1‒14), while the mean age of the patients at coitarche (first sexual intercourse) was 13.86 ± 1.22 years (range: 9‒17 years).

One adolescent (0.33%) presented a High-grade Squamous Intraepithelial Lesion (HSIL) on her Pap smear test, while 29 (9.57%) presented a Low-grade Squamous epithelial Lesion (LSIL) and 23 (7.59%) presented Atypical Squamous Cells of Undetermined Significance (ASC-US), totaling 17.49% of abnormal Pap smears.

From the demographic perspective, a comparison between the subgroup of oncogenic HPV patients and those without HPV shows that the patients with viral detection had comparatively little schooling (p = 0.02) and were single (OR = 2.15, 95% IC 1.14‒4.06, p = 0.02) (Table 1). There was no statistically significant difference between pregnant adolescents with HPV and those without a diagnosis of HPV regarding variables related to gynecological and obstetric history (Table 2).

**Table 1:** Association between sociodemographic variables and oncogenic HPV-caused infection in pregnant adolescents– univariate analysis.

Characteristics	Oncogenic HPV		OR (95% CI)	p-value
	Positive (Mean ± SD or %)	Negative (Mean ± SD or%)		
Age (years)	15.18(±1.33)	15.41(±1.09)		0.099
Partner's age (years)	20.56(±4.35)	20.33(±4.08)		0.637
Schooling (years of study)	8.84(±1.74)	9.32(±1.81)		0.021^b^
Marital status				0.018^b^
Stable relationship or single with partner	120 (78.43)	133 (88.67)	1	
Single without partner	33 (21.57)	17 (11.33)	2.15 (1.14‒4.06)	
Religion				
Catholic	68 (44.74)	66 (44)	1	
Evangelical	55 (36.18)	46 (30.67)	1.16 (0.69‒1.95)	0.573
None	24 (15.79)	34 (22.67)	0.69 (0.37‒1.28)	0.233
Other	5 (3.29)	4 (2.67)	1.21 (0.31‒4.72)	0.780
Practice of religion				
No	104 (68)	96 (64)	1	
Yes	49 (32)	54 (36)	0.84 (0.52‒1.35)	0.465
Family income				
< 2 MS	34 (22.52)	35 (23.49)	1	
Betweeen 2 and 5 MS	47 (31.13)	49 (32.89)	0.99 (0.53‒1.83)	0.967
> 5 MS	20 (13.25)	11 (7.38)	1.87 (0.78‒4.49)	0.159
Not informed	50 (33.10)	54 (36.24)	0.95 (0.52‒1.75)	0.877
Alcohol consumption before pregnancy				
No	133 (86.93)	138 (92)		
Yes	20 (13.07)	12 (8)	1.73 (0.81‒3.68)	0.149
Smoking before pregnancy				
No	132 (86.27)	139 (92.67)		
Yes	21 (13.73)	11 (7.33)	2.01 (0.93‒4.33)	0.068
Marijuana use before pregnancy				
No	138 (92)	141 (92.16)	1	
Yes	12 (8)	12 (7.84)	1.02 (0.44‒2.35)	0.96
Use of cocaine before pregnancy				
No	149 (97.39)	150 (100)		
Yes	4 (2.61)	0 (0)	9.06 (0.48‒169.7)	0.140
				

^a^The numbers do not always add up to the total due to missing values.

SD, Standard Deviation; OR, Odds Ratio; CI, Confidence Interval; MS, Minimum Salary.

b Statistically significant value.

**Table 2:** Association between gynecological and obstetric history and oncogenic HPV-caused infection in pregnant adolescents^a^– univariate analysis.

Characteristics	Oncogenic HPV		OR (95% CI)	p-value
	Positive (Mean ± SD or %)	Negative (Mean ± SD or %)		
Visits to the gynecologist before pregnancy				
No	88 (57.52)	73 (48.67)	1	
Yes	65 (42.48)	77 (51.33)	0.70 (0.44‒1.10)	0.122
CM used before pregnancy				
None	41 (26.97)	37 (24.67)	1	
Coitus interruptus	2 (1.32)	0 (0)	4.52 (0.21‒97.2)	0.335
Hormonal	41 (26.97)	44 (29.33)	0.84 (0.45‒1.56)	0.581
Barrier (condom)	68 (44.74)	69 (46)	0.89 (0.51‒1.55)	0.679
Adequate use of CM before pregnancy				0.615
No	119 (77.8)	113 (75.3)	1	
Yes	34 (22.2)	37 (24.7)	0.87 (0.51‒1.49)	
Adequate use of male condom before pregnancy				0.785
No	132 (86.3)	131 (87.3%)	1	
Yes	21 (13.7)	19 (12.7)	1.10 (0.56‒2.14)	
Coitarche	13.8 (±1.26)	13.9 (±1.23)		0.409
Number of partners in her lifetime	2.07 (±1.98)	1.71 (±1.26)		0.068
Frequency of sexual intercourse (per week)	1.55 (±1.45)	1.93 (±2.06)		0.084
Time span of sexual relations before pregnancy (in months)	17.7 (±12.8)	17.8 (±13.3)		0.923

a The numbers do not always add up to the total due to missing values. SD, Standard Deviation; OR, Odds Ratio; CI, Confidence Interval; CM, Contraceptive Method.

Of the total of 303 adolescents evaluated in the study, only 289 had information about cervicovaginal cytology. Of these 289 pregnant women, 147 were diagnosed with oncogenic HPV and 142 were not diagnosed with oncogenic HPV. Of the adolescents diagnosed with high-risk HPV, 29.93% (44/147) presented cervical cytological alterations, while only 6.34% (9/142) of the adolescents without high-risk HPV presented cytological alterations in the cervix. The diagnosis of cervical oncogenic HPV was associated with both the ASC-US (OR = 5.13, 95% CI 1.71‒16.54) and the LSIL (OR = 9.23, 95% CI 2.80‒30.39) findings. Although there was only one HSIL case, this one case appeared to be related to the presence of oncogenic HPV (Table 3).

**Table 3:** Association between clinical and laboratory characteristics and oncogenic HPV-caused infection in pregnant adolescents^a^– univariate analysis.

Characteristics	Oncogenic HPV		OR (95% CI)	p-value
	Positive (Mean ± SD or %)	Negative (Mean ± SD or %)		
Weight before pregnancy	54.30 (±9.85)	54.66 (±10.4)		0.767
BMI before pregnancy	21.32 (±3.08)	21.57 (±3.77)		0.560
Secretion				0.744
Others	107 (87)	99 (88.4)	1	
Greenish, yellowish, or grayish	16 (13.0)	13 (11.6)	1.14 (0.52‒2.49)	
Result of OCC collected at prenatal care				
Normal	21 (14.29)	31 (21.83)	1	
Benign cell changes	82 (55.78)	102 (71.83)	1.19 (0.63‒2.22)	0.591
HSIL	1 (0.69)	0 (0)	4.40 (0.17‒113.1)	0.371
LSIL	25 (17.0)	4 (2.82)	9.23 (2.80‒30.39)	<0.001^b^
ASC-US	18 (12.24)	5 (3.52)	5.31 (1.71‒16.54)	0.004^b^

a The numbers do not always add up to the total due to missing values. SD, Standard Deviation; OR, Odds Ratio; CI, Confidence Interval; BMI, Body Mass Index; OCC, Oncotic Colpocytology; HSIL, High-grade Squamous Intraepithelial Lesion; LSIL, Low-grade Squamous Intraepithelial Lesion; ASC-US, Atypical Squamous Cells of Undetermined Significance.

b p-value, Statistically significant value.

When the authors evaluated the clinical characteristics of pregnant women, such as preconception weight and body mass index before pregnancy, we also did not observe a statistically significant difference between adolescents with HPV and those without a diagnosis of HPV (Table 3). When the authors evaluated the laboratory characteristics of the pregnant women, we observed that there was no statistically significant difference between the groups of adolescents regarding the microbiology profile observed in oncotic colpocytology (Table 3).

As for obstetric outcomes, 55.21% of the overall group of pregnant adolescents underwent vaginal delivery without an instrument and 30.21% underwent a cesarean section. Interestingly, the oncogenic HPV detection did not interfere with the type of delivery nor with the neonatal result as shown in Table 4.

**Table 4:** Association between obstetric outcomes and oncogenic HPV-caused infection in pregnant adolescents^a^ – univariate analysis.

Characterístics	Oncogenic HPV		OR (95% CI)	p-value
	Positive (Mean ± SD or %)	Negative (Mean ± SD or %)		
Type of delivery				
Vaginal	57 (57.58)	49 (52.69)	1	
Cesarean	32 (32.32)	26 (27.96)	1.06 (0.56‒2.01)	0.863
Forceps	10 (10.10)	18 (19.35)	0.48 (0.20‒1.13)	0.093
Premature birth				
No	92 (89.32)	93 (92.08)		
Yes	11 (10.68)	8 (7.92)	1.39 (0.53‒3.61)	0.497
Gestational age at delivery	38.49 (±2.34)	38.90 (±1.68)		0.249
Weight classification of the NB				
AGA	69 (72.63)	70 (76.92)	1	
SGA	23 (24.21)	19 (20.88)	1.23 (0.61‒2.45)	0.561
LGA	3 (3.16)	2 (2.20)	1.52 (0.25‒9.39)	0.651
Weight at birth	3010.01 (±580.82)	3105.99 (±558.42)		0.243
1-min APGAR mean score	8.60 (±1.16)	8.46 (±1.82)		0.538
APGAR score < 7 at 1-min				
No	82 (93.2)	78 (91.8)	1	
Yes	6 (6.8)	7 (8.2)	0.82 (0.26‒2.53)	0.724
5-min APGAR mean score	9.53 (±0.93)	9.20 (±1.56)		0.096
APGAR score < 7 at 5-min				
No	88 (98.9)	1		
Yes	1 (1.1)	5 (5.9)	0.18 (0.02‒1.59)	0.123
Stay in nursery (days)	5.99 (±10.01)	4.08 (±3.96)		0.374
Number of prenatal care visits	7.67 (±2.80)	8.18 (±2.64)		0.205

a The numbers do not always add up to the total due to missing values. SD, Standard Deviation; OR, Odds Ratio; CI, Confidence Interval; BMI, Body Mass Index; NB, Newborn; AGA, Appropriate for Gestational Age; SGA, Small for Gestational Age; LGA, Large for Gestational Age.

A multivariate analysis of the data was carried out to determine the risk factors independently associated with cervical detection of oncogenic HPV. Schooling (years of study) turned out to be an independent factor for protection against the cervical diagnosis of high-risk HPV (p = 0.03, OR = 0.85, 95% CI 0.73‒0.99). The number of sexual partners in the patient's lifetime (p = 0.03, OR = 1.27, 95% CI 1.03‒1.56) and having religion as opposed to not having one (p = 0.03, OR = 2.05, 95% CI 1.08‒3.92) were also shown to be independent risk factors. In the final model, adjustments were made for weekly frequency of sexual intercourse and marital status (single with or without a partner, married, or informally married). This model proved to be highly adequate to predict the final event (p = 0.92 on the Hosmer and Lemeshow test). The other variables were not independent predictors of a diagnosis of cervical high-risk HPV (Table 5).

**Table 5:** Association between epidemiological variables and oncogenic HPV-caused infection in pregnant adolescents – multivariate analysis.

	Significance	OR adjust	95% CI	
			Lower	Upper
Years of schooling	0.03	0.85	0.73	0.99
Number of sexual partners in lifetime	0.03	1.27	1.03	1.56
Religion (any religion vs. no religion)	0.03	2.05	1.08	3.92
Sexual intercourse frequency per week	0.08	0.87	0.75	1.02
Marital status	0.22	1.62	0.75	3.48
Constant	0.41	1.89		

Model adjusted for sexual intercourse frequency per week, and marital status (single with a partner, or single without a partner vs. married or informally married). Hosmer and Lemeshow Test: p = 0.91.

HPV, Human Papillomavirus; OR, Odds Ratio; CI, Confidence Interval.

## Discussion

This study reported a high-risk oncogenic cervical HPV-DNA frequency of 50.50% among its 303 pregnant adolescents. In the literature, the prevalence of oncogenic HPV among pregnant women ranges from 5.80% to 51.70%.^20^, ^21^, ^22^, ^23^, ^24^ A study that included 1016719 women with normal cytological findings identified an overall prevalence of cervical HPV infection of 11.7%, with the highest prevalence being found in Sub-Saharan Africa (24%), Eastern Europe (21.4%), and in Latin America (16.2%).^25^ This study demonstrated that the distribution of cervical HPV infection has a first peak at younger ages (< 25 years) and a rebound at older ages (≥ 45 years).^25^ A meta-analysis of 28 studies identified a prevalence of cervical infection by HPV of 23.94% in pregnant women under 25 years of age and a frequency of 18.00% in non-pregnant women of the same age group, demonstrating a statistically significant difference (p = 0.03).^26^ This meta-analysis concluded that there was no statistically significant difference between pregnant and non-pregnant women regarding the prevalence of different types of HPV and also showed that pregnant women have a significantly increased risk of HPV infection when compared to non-pregnant women (OR = 1.42, 95% CI 1.25‒1.61), especially those younger than 25 years old (OR = 1.79, 95% CI 1.22‒2.63).^26^

The prevalence found in the present study does not seem to differ from the rate observed at the same clinic ten years previously, that of 51.70%, in a study with 60 pregnant adolescents.^20^ Nevertheless, in the medical literature, the frequency of high-risk HPV on the cervix among adult women tends to be lower than that found among adolescents in the present study.^7^^,^^10^^,^^15^^,^^16^^,^^21^^,^^24^^,^^27^, ^28^, ^29^, ^30^, ^31^, ^32^, ^33^, ^34^

Such a high prevalence of oncogenic HPV among pregnant adolescents prompts us to ponder the risk of not having them undergo a Pap smear as most recently recommended by the Brazilian Ministry of Health. The present policy is to limit the Pap smear to women aged 25 years or older in accordance with a few international protocols. The present study presents rates of colpocytological changes of 17.50%, with 10% of intraepithelial lesions, which are higher than that reported in most of the most recent studies of the Brazilian population, whose frequencies vary between 2.56% and 10.23%.^35^^,^^36^ These are rates not to be overlooked.

There is evidence that favors an earlier approach to cervical cancer screening and others that favor a later collection of oncotic colpocytology. An IARC (International Agency for Research on Cancer) study showed that starting cervical cytopathological screening at age 25, rather than age 20, had a reduction of only 1% in the cumulative incidence of cervical cancer.^37^ Another British study demonstrated overtreatment and a modest benefit of cervical cancer screening at age 20 rather than age 25; to prevent one case of invasive carcinoma, it would be necessary to treat between 300 and 900 women with cervical intraepithelial neoplasia. The authors should also think about the possible obstetric consequences, such as premature birth, and psychological consequences of treating precursor lesions of cervical cancer in young women.^38^^,^^39^ A Canadian study with pregnant adolescents identified a high frequency of low-grade cervical cytological lesions, with a high rate of regression; the screening strategy increased the rate of interventions and did not prevent any cases of cervical cancer.^40^ On the other hand, Wright et al., in a North American retrospective study, identified that the risk of adolescents diagnosed with intraepithelial lesions progressing to high-grade cervical abnormalities was similar to the risk observed in adults.^41^

Certainly, vaccination of preadolescents and adolescents against HPV is an important public health action, which should lead to lower rates of HPV-caused infection and cytopathological abnormalities in such patients. However, despite the fact that government vaccination in Brazil began in March 2014, the infection rates remain high when compared to previous data. In a study carried out with 152 pregnant adolescents in Brazil, not only was low adherence to the vaccination program found (only 44%), but also a remarkable ignorance of the ways to avoid being infected by HPV (66% did not know how HPV was transmitted).^42^ To assess the effects of vaccination programs, a meta-analysis of studies from high-income countries observed that, even in places with female vaccination coverage below 50%, there was a significant reduction in HPV types 16 and 18 infections (RR = 0.50, 95% CI 0.34‒0.74).^43^ Another meta-analysis found that 5 to 9 years after the introduction of immunization, there was an approximately 83% drop in the prevalence of HPV types 16 and 18 among girls aged 13 to 19 years (RR = 0.17, 95% CI 0.11‒0.25), a 54% decrease in the prevalence of HPV types 31, 33 and 45 among girls aged 13 to 19 years (RR = 0.46, 95% CI 0.33‒0,66) and a 51% decrease in the frequency of grade 2 cervical intraepithelial neoplasia among girls aged 15 to 19 years (RR = 0.49, 95% CI 0.42‒0.58).^44^ These data reinforce the need to encourage vaccination among adolescents. Rodriguez et al. show us that behavioral and informational interventions double the frequency of administration of the first dose of immunization and that behavioral interventions increase the frequency of administration of the last dose of vaccine by 68%.^45^

Furthermore, the detection of LSIL in the oncotic colpocytology of pregnant adolescents was strongly associated with the presence of oncogenic HPV (p < 0.001, OR = 9.23, 95% CI 2.80‒30.39) and of ASC-US (p = 0.004, OR = 5.31, 95% CI = 1.71‒16.54). This result is in consonance with the current literature.^6^

No association was observed between the diagnosis of HSIL and the occurrence of high-risk cervical HPV. It was not statistically significant, though, most likely because there was only one case of HSIL among the 303 women assessed in the present study. As it is an infrequent event, a much larger cohort would be necessary to show a statistical connection between oncogenic HPV-caused infection and the presence of HSIL. As a matter of fact, such a link has already been well established in the literature. Another important piece of information is that the adolescent with cervical cytological results of HSIL had systemic lupus erythematosus and progressed to hypertensive pregnancy syndrome and delivery at 25 weeks gestation. Although the institutional protocol guides the performance of colposcopy in all pregnant women with a cytological diagnosis of the high-grade intraepithelial lesion, regardless of age, there was not enough time for further investigation of this adolescent. After delivery, there was loss of gynecological and obstetric follow-up of this patient.

Evaluation of the risk factors associated with the detection of oncogenic HPV-DNA revealed that less schooling, measured as fewer years of study, turned out to be an independent risk factor for the disease in pregnant adolescents. There is similar evidence in the literature.^8^^,^^10^ Zhang et al., evaluating 10,000 volunteers from Shanghai, observed an association between higher educational levels and lower rates of cervical HPV positivity (p = 0.045).^8^ Quan-Fuma et al., evaluating 1858 women from Hubei, found no association between schooling and high-risk oncogenic cervical HPV infection.^46^ Kliucinskas et al., in a prospective cohort that included 1120 women from 8 health institutions in Lithuania, also observed that a lower educational level (high school or lower) was related to an increase in the prevalence of high-risk HPV (OR = 1.43, 95% CI 1.01‒2.04).^10^

A large number of partners in an adolescent's lifetime was also identified as a predictor of cervical detection of oncogenic HPV. There is evidence in the literature to corroborate this finding.^8^^,^^11^^,^^12^^,^^32^^,^^34^ Zhang et al., evaluating a total of 10,000 patients, concluded that the number of sexual partners greater than or equal to 2 was associated with the diagnosis of cervical HPV infection (p = 0.001).^8^ Rio-Ospina et al., evaluating the frequency of 6 high-risk oncogenic HPV subtypes in a total of 2134 Colombian women aged 12 to 19 years, observed that a history of 3 or more sexual partners during their lifetime was associated with the diagnosis of cervical HPV infection (OR = 1.77, 95% CI 1.11‒2.81).^11^ And when analyzing 312 North American urban adolescents, Tarkowski et al. concluded that the greater the number of sexual partners during life, the greater the risk of cervical HPV infection; in the multivariate analysis, the number of sexual partners greater than or equal to 8 had an odds ratio of 7.4 (95% CI 2.9‒18.5).^12^

The practice of religion stood out as well as an independent risk factor for high-risk cervical HPV. There are no similar reports in the literature. This finding may be linked to the fact that women with religious beliefs show a behavioral pattern that differs from that of women with no such beliefs. However, one would imagine, at least in theory, that religion would encourage healthy behaviors and self-care, an expectation that runs counter to the findings of this research.

One might say that adolescents with religious beliefs are at a greater disadvantage in terms of socioeconomic status. But this was not the case. These girls had a higher mean of school years (p = 0.009, 9.20 vs. 8.55 years) and higher rates of family income, which was over two minimum salaries (p = 0.05, OR = 2.27, 95% CI 1.06‒4.84). Besides, most of the adolescents who declared a belief in religion denied they actually practiced it (141/244 = 57.78%), These findings, therefore, add to the uncertainty about the factors which could explain the association between religious beliefs and cervical detection of high-risk oncogenic HPV-DNA.

With regard to the pattern of sexual activity of young Christian women, Brooks et al. observed that the more religious a young woman is, the less likely she is to be sexually active; however, when initiating sexual intercourse, the probability of using condoms is lower.^47^ Koletic et al. also observed a small association between the profession of a religion and a lower number of sexual partners during life and older age of coitarche; however, they did not observe an association between religiosity and condom use.^48^ Additional qualitative studies might provide a better assessment of the reasons for such a connection.

Additionally, being a single pregnant woman without a steady partner was also a risk factor in univariate analysis (p = 0.02, OR = 2.15, 95% CI 1.14‒4.06); however, this was not confirmed by the multivariate analysis (p = 0.14, OR = 1.65, 95% CI 0.85‒3.22). This outcome may have resulted from the fact that patients without a stable relationship tend to have a pattern of sexual activity which is different from that which has been observed in adolescents with a steady partner.^7^^,^^29^^,^^32^ The literature reinforces this finding of a higher frequency of cervical HPV among single people when compared to married people, as in the studies by Ronco et al. (OR = 2.23, 95% CI 1.28‒3.89), from Thomas et al. (OR = 2.1, 95% CI 1.1‒3.9) and from Giuliano et al. (aOR = 1.9).^7^^,^^29^^,^^49^ Branstetter et al. also identified that the fact that the woman was married or cohabiting reduced the risk of cervical HPV infection (aOR = 0.62, 95% CI 0.44‒0.89).^32^

None of the obstetric outcome variables turned out to be statistically significant when the groups were compared. An Israeli population-based retrospective cohort also found no association between cervical HPV infection and unfavorable obstetric outcomes, such as premature rupture of ovular membranes, small-for-gestational-age newborns, hypertensive pregnancy syndrome, or premature placental abruption.^50^ However, other studies demonstrate an association between cervical HPV infection and the occurrence of some adverse obstetric outcomes such as premature birth, preterm premature rupture of membranes, intrauterine growth restriction, low birth weight, and fetal death.^51^^,^^52^^,53^ A meta-analysis of 36 studies concluded that cervical HPV infection was associated with preterm delivery (aOR = 1.50, 95% CI 1.19‒1.88), premature rupture of ovular membranes (aOR = 1.42, 95% CI 1.08‒1.96), with intrauterine growth restriction (aOR = 1.17, 95% CI 1.01‒1.37), with low birth weight (aOR = 1.91, 95% CI 1.33‒2.76) and with fetal death (aOR = 2.23, 95% CI 1.14‒4.37).^51^

Since this was a hospital-based study, the study sample may not correspond exactly with the general community. Another point that may be considered a limitation is a fact that the hospital where this study was carried out assists primarily women of low socioeconomic status, thus preventing the results from being generalized to the overall population. As it was a retrospective study, there were significant data losses on some variables related to obstetric and neonatal outcomes, limiting the analysis of the results. The type of research carried out allows only raising hypotheses, requiring prospective and intervention studies to confirm the findings.

However, the present findings confirm the relevance of the topic, indicating the need for educational measures to reduce the incidence of sexually transmitted infections in adolescents and to strengthen adherence to HPV vaccination programs.

## Conclusions

High-risk oncogenic cervical HPV-DNA was detected in more than half of the pregnant adolescents analyzed in the present study. Less schooling, a larger number of partners in their lifetime, and having a religion were shown to be independent risk factors for a cervical infection caused by high-risk HPV among adolescents. In the univariate analysis, being a pregnant adolescent without a steady partner turned out to be a risk factor for oncogenic cervical HPV. Obstetric outcomes, such as premature delivery, newborn weight at birth, APGAR score, number of days of hospitalization of the neonate, umbilical cord pH, or number of prenatal consultations, were not statistically different between the groups. However, studies with larger samples are needed to achieve more consistent results. The LSIL and the ASC-US colpocytological findings were strongly associated with high-risk cervical HPV. The LSIL, HSIL, and ASC-US rates among adolescents were, respectively, 9.57%, 0.33% and 7.59%, totaling 17.49% of cytological abnormalities. Such findings point to the need for additional qualitative studies to determine more precisely the type of behavior involved and the best way to address the risk associated with HPV-caused cervical infection during adolescence.

Despite the high frequency of oncogenic HPV in the pregnant adolescent population, the literature shows a high tendency towards clearing cervical infections and a high rate of regression of cervical lesions. However, there is still a lack of information about the influence of the pregnancy period on the evolution of intraepithelial lesions in women under 20 years of age, requiring additional investigations. The present study also made it possible to identify adolescents at greater risk of acquiring sexually transmitted diseases, facilitating the orientation of possible health programs. Informational interventions to encourage vaccination can be presented as a solution to reduce the frequency of cervical infection and decrease the sexual transmission of oncogenic HPV in adolescents.

## Ethics approval and consent to participate

The Research Ethics Committee of the Hospital das Clínicas da Faculdade de Medicina da Universidade de São Paulo approved the present study (presentation certificate for ethical appraisal number 65511617.0.0000.0068).

## Consent for publication

Authors accept the journal's rules for publication and assign their copyright to the publisher.

## Availability of data and material

The authors will make available the research data, according to personal request.

## Funding

The authors declare that they have not received any form of funding.

## Declaration of Competing Interest

The authors declare that they have no conflicts of interest for the subject of this publication. None of the authors received any benefits, financial or non-financial interests, directly or indirectly related to the work submitted for publication.
